# Supplementary material for: Evolutionary Genetic Analysis Uncovers Multiple Species with Distinct Habitat Preferences and Antibiotic Resistance Phenotypes in the Stenotrophomonas maltophilia Complex
Source: Front Microbiol. 2017 Aug 17;8:1548. doi: 10.3389/fmicb.2017.01548 (PMC5562727; doi:10.3389/fmicb.2017.01548)
Supplement: Supplementary file 1 [file Presentation_1.pdf]

Evolutionary genetic analysis uncovers multiple species with distinct habitat preferences and antibiotic resistance phenotypes in the *Stenotrophomonas maltophilia* complex

Luz Edith Ochoa-Sánchez and Pablo Vinuesa*
Centro de Ciencias Genómicas, Universidad Nacional Autónoma de México, Cuernavaca, Morelos, Mexico.

***Correspondence:**
Corresponding Author
vinuesa@ccg.unam.mx

Keywords: *Stenotrophomonas maltophilia* complex taxonomy, antibiotic resistance, multidrug resistance, multispecies coalescent, population genetic structure, recombination, metallo-beta-lactamase, niche partitioning.

Running title: *Stenotrophomonas* evolutionary genetics and ecology

Supplementary materials

Frontiers in Microbiology | August 2017 | Volume 8 | Article 1548

http://journal.frontiersin.org/article/10.3389/fmicb.2017.01548/

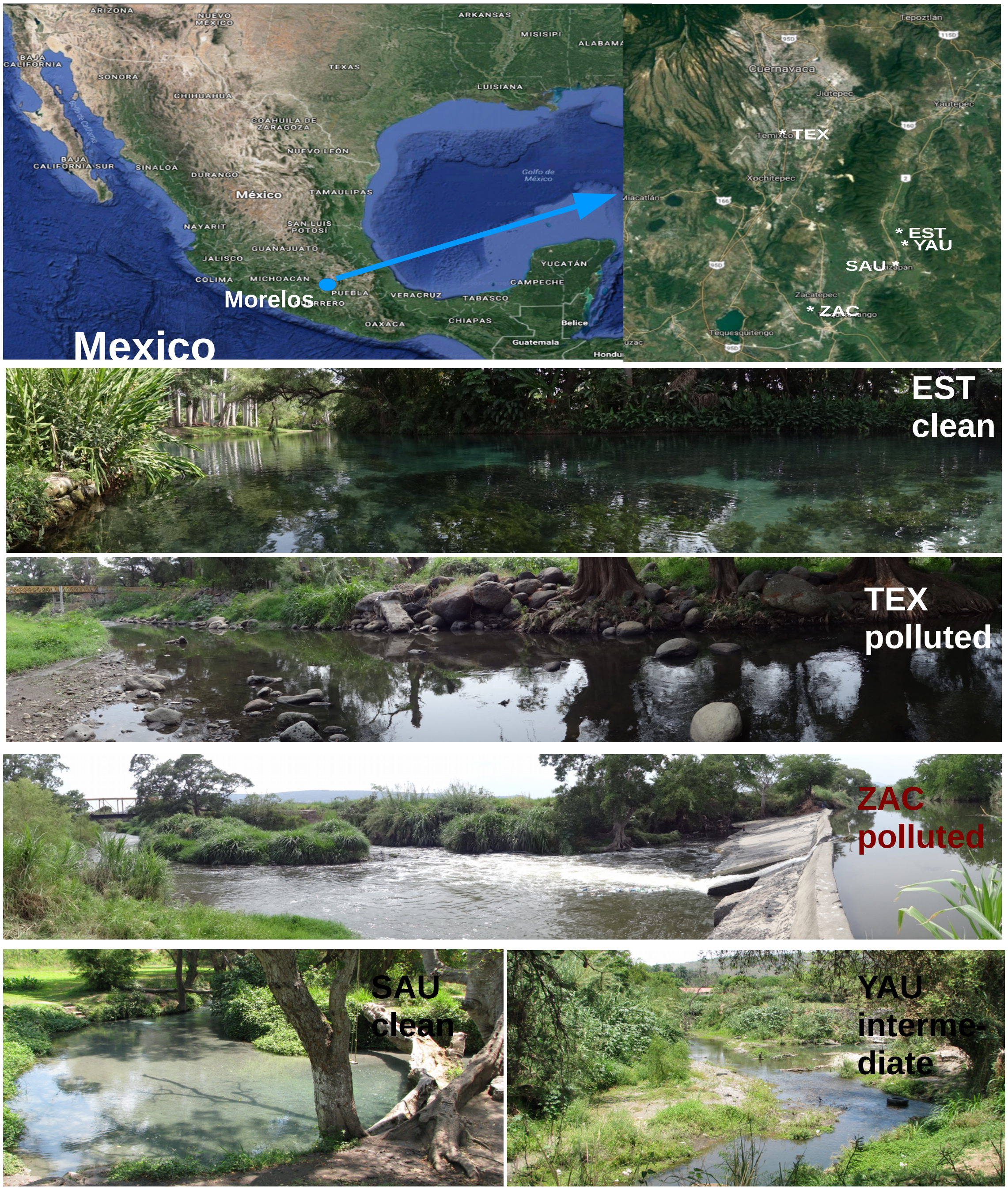

Figure S1. Maps and pictures showing the location and aspect of the 6 sampling sites in 4 rivers and streams in Morelos, Central Mexico, analyzed in this work. The coordinates of the sites and other characteristics are presented in Table 1 of the main text.

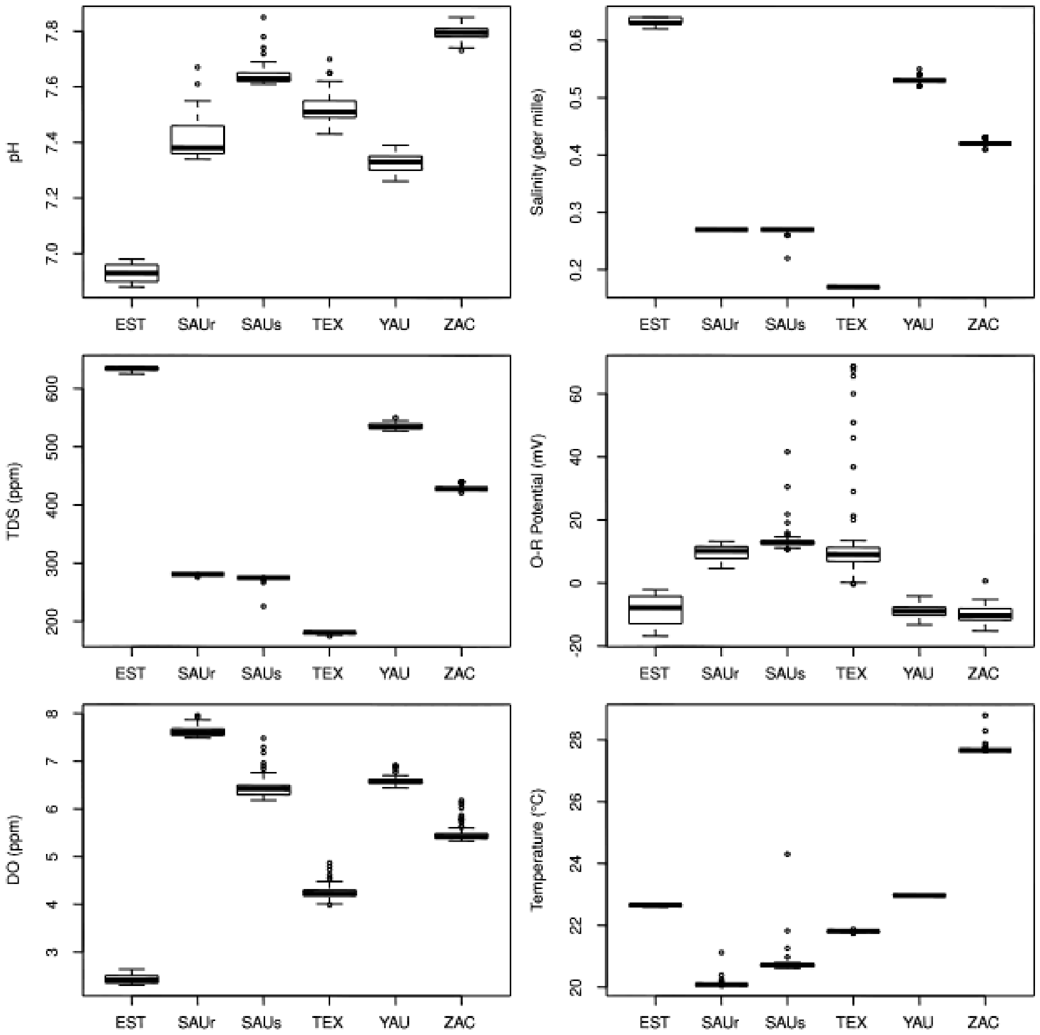

Figure S2. Parallel boxplots summarizing the distribution of values for 6 environmental parameters measured at the 6 sampling sites. The measurements for each site were taken with a HANNA multi-parametric HI9828 instrument operated in continuous measurement mode for 1 min, along a 10 m transect at each sampling site. (TDS = total dissolved solutes; O-R = oxido-reduction; DO = dissolved oxygen).

Plate antibiotic resistance profiling and CLSI break points

Table S1. CLSI 2016 breakpoints for plate assays.

**Antimicrobial Agent[a]**<br>*This work (µg /ml)	**Break points CLSI 2016 (plate assays)<br>Interpretative criteria (concantrations in µg/mL)[b]**								
	Pseudomonas aeruginosa			***Stenotrophomonas maltophilia***			***Enterobacteriaceae***		
	**S**	**I**	**R**	**S**	**I**	**R**	**S**	**I**	**R**
**1.- Beta-lactams**									
**a) Cephems:**									
Ceftazidime 16	≤8	16	≥32	≤8	16	≥32	≤4	8	≥16
Cefotaxime 4							≤1	2	≥ 4
**b) Carbapenems:**									
Imipenem 4	≤2	4	≥8				≤1	2	≥4
**c) Beta-lactamase/ inhibitor combinations:**									
Piperacillin-tazobactam 128/4	≤16/4	32/4-64/4	≥128/4				≤16/4	32/4-64/4	≥128/4
**2.- Aminoglycosides:**									
Gentamicin 16	≤4	8	≥16				≤4	8	≥16
Kanamycin 64							<16	32	>64
Streptomycin 50							*There are no MIC interpretative standards		
**3.- Phenicols:**									
Chlorampheinicol 30				≤8	16	≥32	≤8	16	≥32
**4.- Quinolones:**									
Nalidixic acid 32							≤16	–	≥32
**Fluoroquinolones:**									
Ciprofloxacin 4	≤1	2	≥4				≤1	2	≥4
**5.- Tetracyclines:**									
Tetracycline 16							≤4	8	≥16
**6.- Folate Pathway nhibitors:**							Trimethoprim		
Trimethoprim 30 + carbenicilin 100							<8	–	≥16

[a]Antimicrobials assayed and their classification in families, as used in this work.
[b]breakpoints for plate assays currently not available for *Stenotrophomonas* in CLSI 2016 were taken from *Pseudomonas aeruginosa* and *Enterobacteriaceae*.

Double disk synergism (DDS) assay for β-lactamase expression phenotyping and CLSI interpretative criteria for inhibition zones

Briefly, seven antibiotic discs [meropenem (10 µg), imipenem (10 µg), ertapenem (10 µg), aztreonam (30 µg), ceftazidime (30 µg), ceftazidime-clavulanic acid (30/4 µg) and cefepime (30 µg)] (AB Bodish, Solna, Sweden) and one disc with 0.5 M EDTA pH 8.0 (5 µg) were placed on a lawn of each strain grown on Mueller-Hinton plates. These were incubated at 30°C for 24-48 hrs, measuring the diameter of growth-inhibition halos around disks (Table S2), and recording synergisms between them.

Table S2. CLSI 2016 breakpoints for disk-diffusion assays.

**Antimicrobial Agent**<br>*This work (µg)	**Break points CLSI 2016 (disc-diffusion assays) Inhibition zone diameter Interpretative criteria (nearest whole mm)[a]**					
	Pseudomonas aeruginosa			***Enterobacteriaceae***		
	**S**	**I**	**R**	**S**	**I**	**R**
**1.- Beta-lactams**						
**a) Cephems:**						
ceftazidime 30	≥18	15-17	≤14	≥21	18-20	≤17
cefotaxime 30				≥26	23-25	≤22
cefepime 30	≥18	15-17	≤14	≥25	–	≤18
**b) Carbapenems:**						
imipenem 10	≥19	16-18	≤15	≥23	20–22	≤19
meropenem 10	≥19	16-18	≤15	≥23	20–22	≤18
ertapenem 10				≥22	19–21	≤18
**c) Beta-lactamase inhibitor combinations:**						
ceftazidime/cavulanate 30/10						
**2.- Monobactams**<br>aztreonam 30	≥22	16-21	≤15	≥21	18-20	≤17

[a]Breakpoints for disk-diffusion assays are currently not available for *Stenotrophomonas* in CLSI 2016. Therefore we used the breakpoints for *Pseudomonas aeruginosa* and *Enterobacteriaceae*.

Supplementary protocol 1: PCR amplification of 16S rDNA sequences and their phylogenetic analysis

Only the forward strand was sequenced for each amplicon with the fD1 primer, using capillary Sanger-sequencing commercially performed at Macrogen (South Korea). Raw reads were trimmed based on

Phred scores using phred (Ewing et al., 1998) and classified at the genus level using an in-house pipeline programmed in Perl that queries each sequence via blastn (Camacho et al., 2009) against a local instance of high quality, nearly full-length *rrs* sequences of bacterial type strains downloaded from the RDP-II server (Cole et al., 2007), retrieving the five closest hits. A local instance of pre-aligned sequences for this same *rrs* dataset downloaded from the RDP-II server was used to generate multiple sequence alignments of the newly generated sequences and selected blastn hits using the sequence to profile alignment function implemented in clustalw2 (Larkin et al., 2007). The resulting alignment was subjected to maximum-likelihood phylogenetic analysis with phyml 3 (Guindon et al., 2010), under best-fitting substitution models selected by jModelTest2 (Darriba et al., 2012).

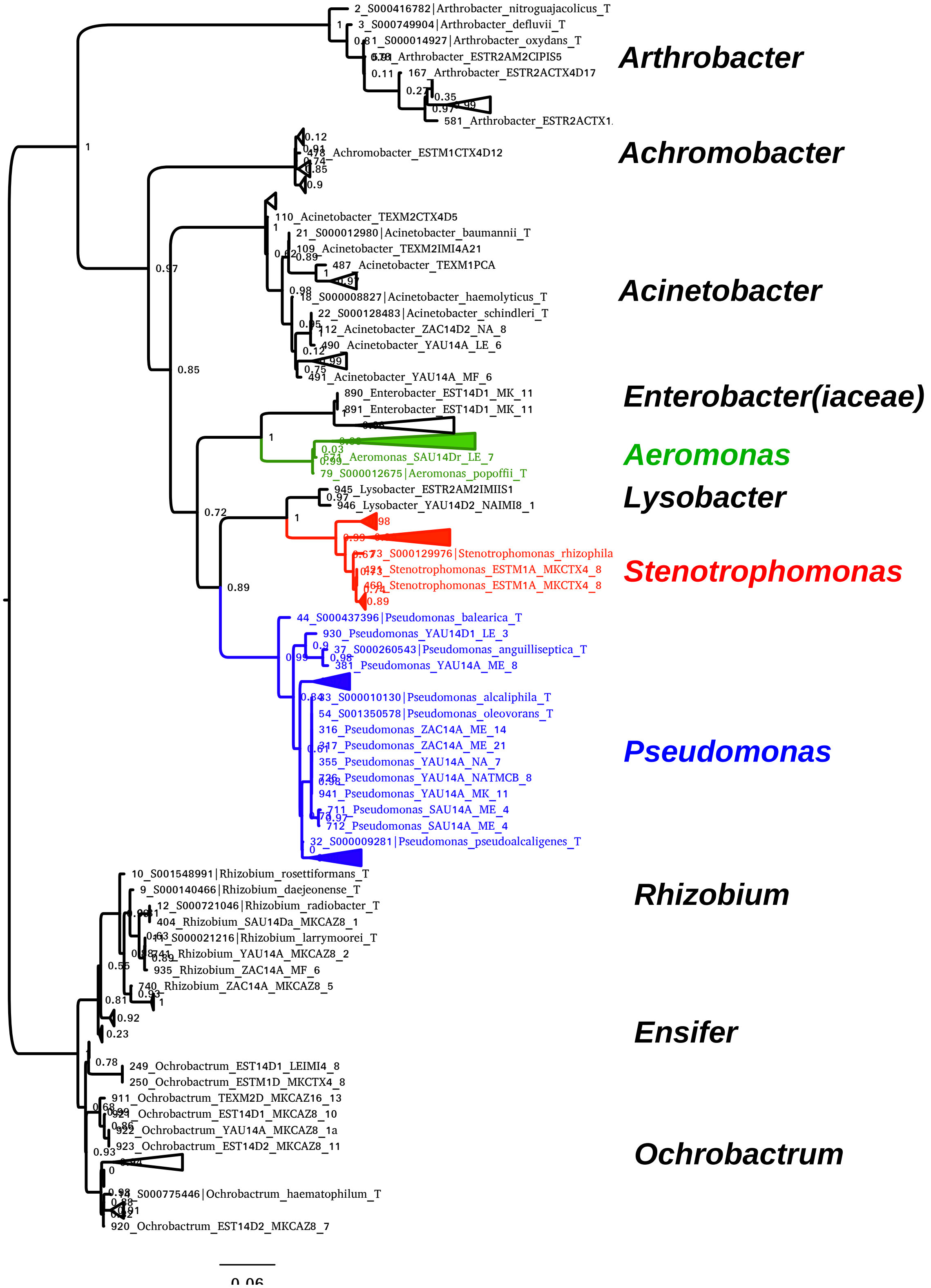

Figure S3. Classification of 697 environmental isolates at the genus level based on maximum likelihood phylogeny of 16S rRNA sequences. Support values on the branches are Shimodaria-Hasegawa-like approximate likelihood ratio test values. Large clades with many sequences are collapsed. Bar represents the expected number of substitutions per site under the GTR+G model.

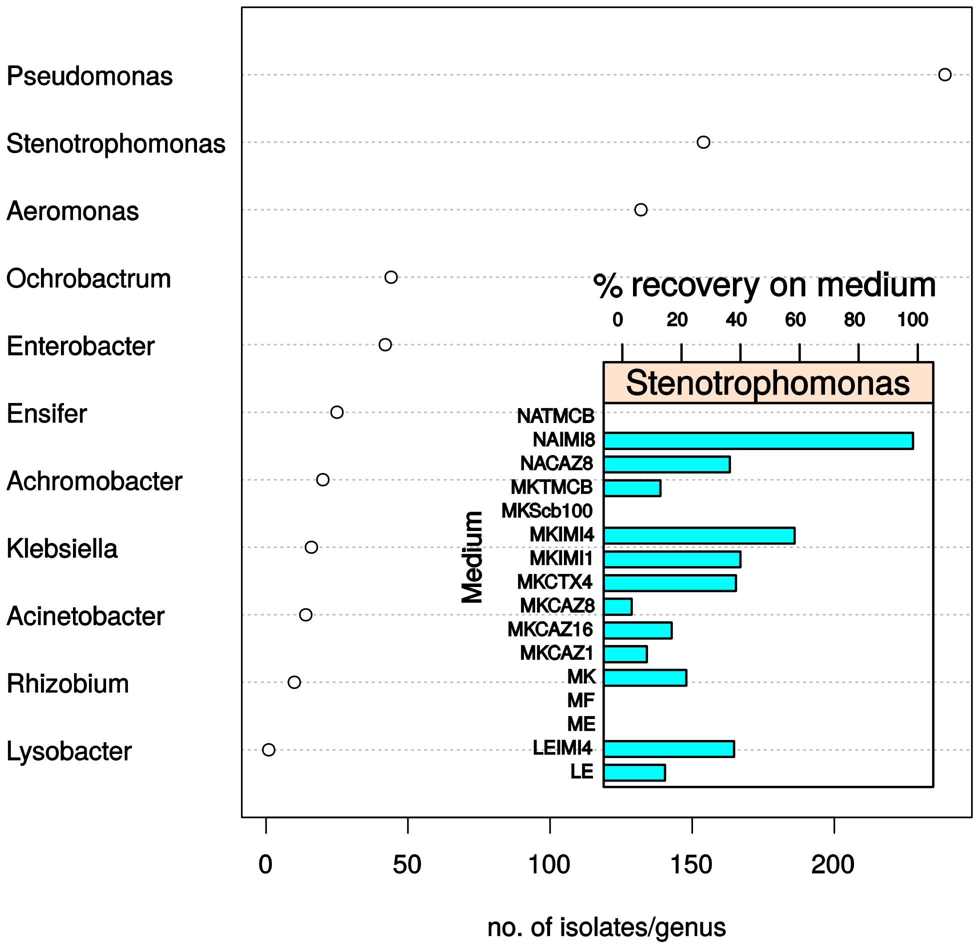

Figure S4. Recovery statistics for different genera (dotplot) and for *Stenotrophomonas* (inset barplot) on the different media amended or not with antibiotics (μg/ml). The media used were: NA = NAA; MK = MacConkey; MF MF=mFC; ME = mTEC; LE = Leeds LAM medium, which are described in the main text. A total of 697 strains were sequenced, and 22.1% of them corresponded to *Stenotrophomonas* spp. based on the 16S phylogenetic classification shown in Fig. S3.

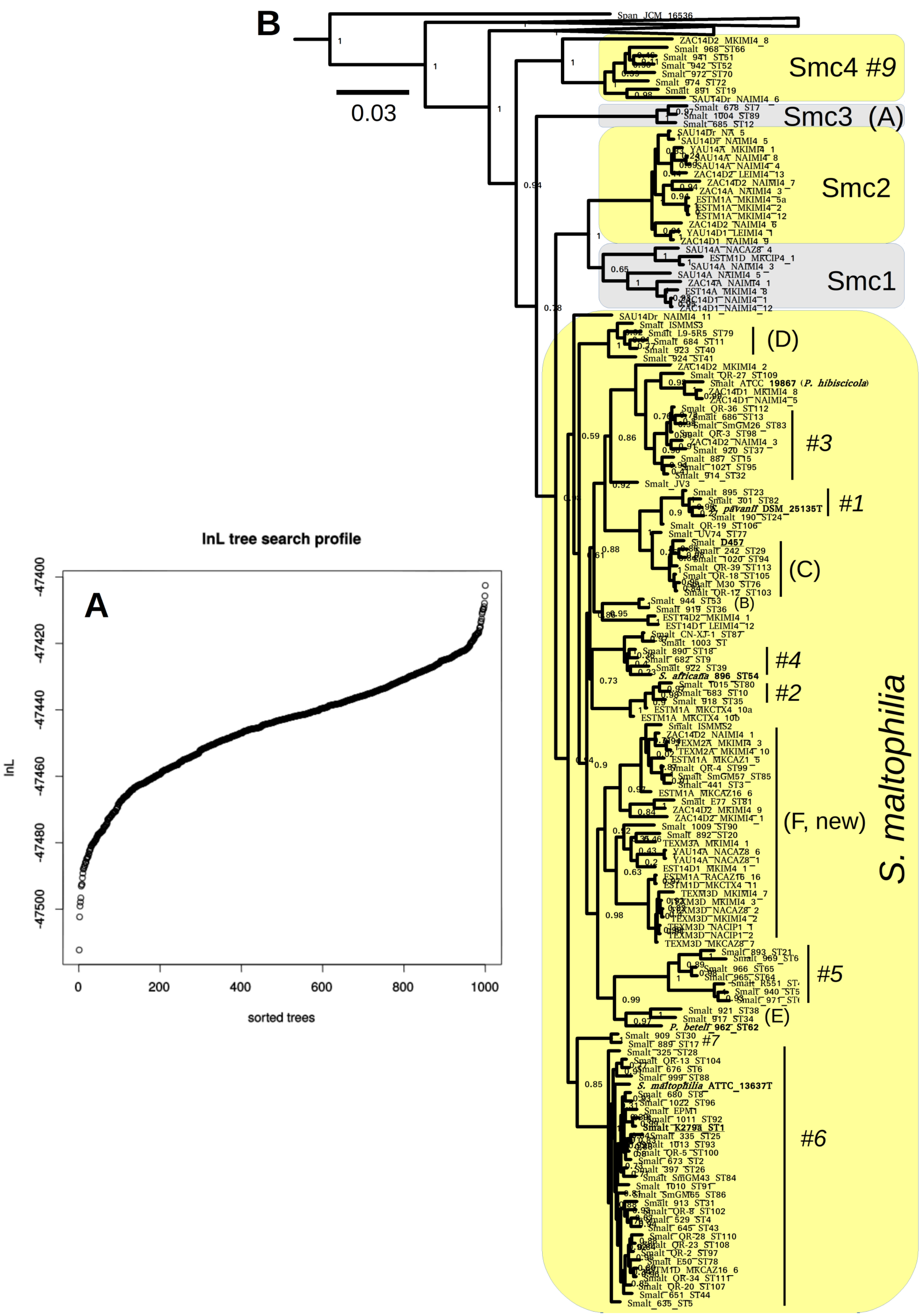
A
lnL tree search profile
lnL
-47400
-47420
-47440
-47460
-47480
-47500
0
200
400
600
800
1000
sorted trees
B
0.03
Smc4 #9
Smc3 (A)
Smc2
Smc1
S. maltophilia
(D)
#3
#1
(C)
(B)
#4
#2
(F, new)
#5
(E)
#7
#6

Figure S5. Results of the maximum-likelihood tree search. **A)** Sorted log-likelihood profile for the 10001 tree searches conducted under the maximum-likelihood criterion, starting from 1000 random trees and 1 BioNJ tree. The top-scoring phylogeny of this search is presented in Figures 1A and 1B in the main text. The ln*L* range = (-47512,-47403). The search started from the BioNJ tree reached a score of -47478.6287. **B)** The same tree as shown in Fig. 1B of the main text, but without collapsing the terminal clades containing only reference strains.

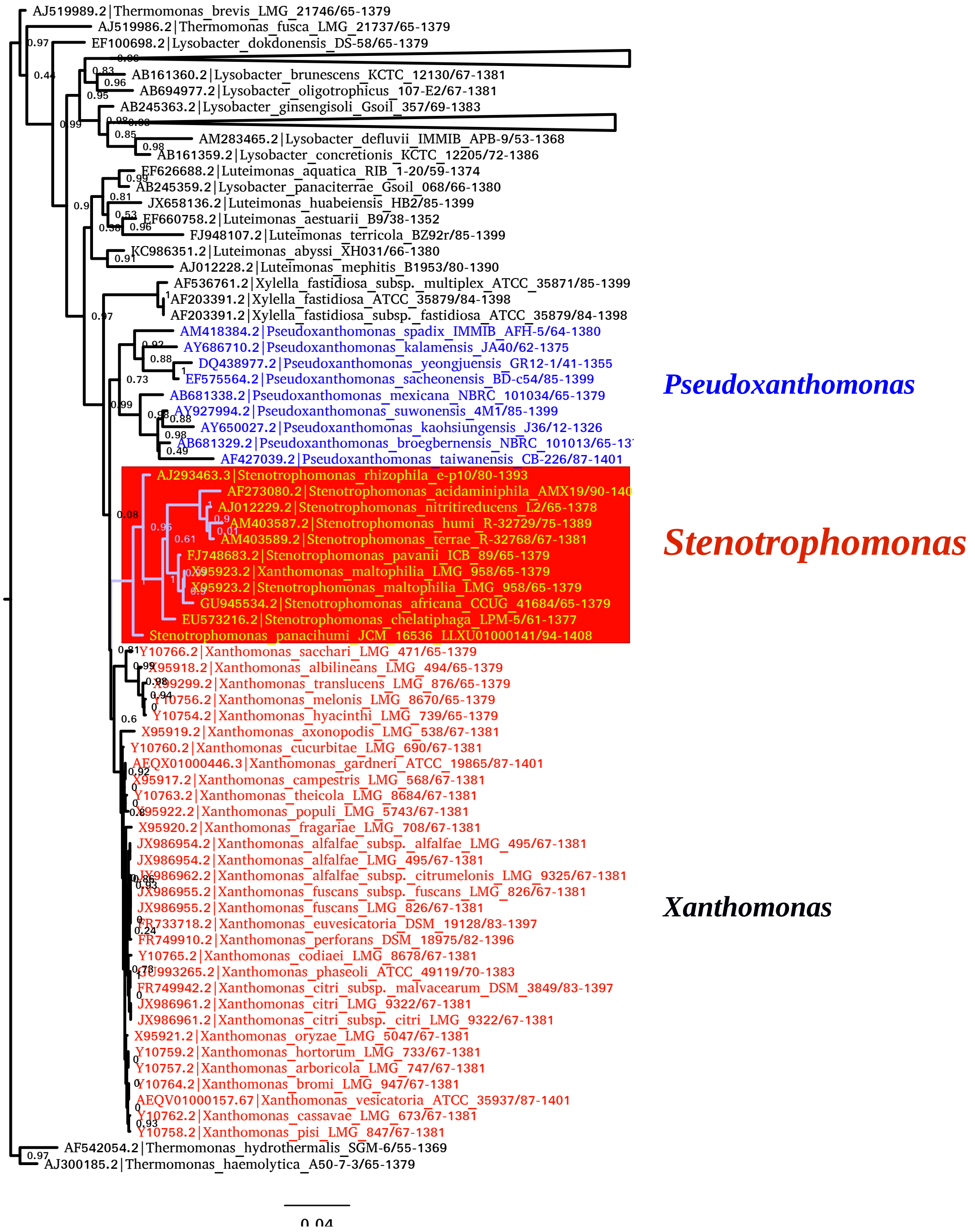

Figure S6. Maximum likelihood phylogeny of nearly full-length 16S rRNA gene sequences corresponding to the type strains of all species in the order *Xanthomonadales*. Some clades distantly related to the focal ones were collapsed to improve the legibility of the leave labels.

Supplementary protocol 2: Bayesian stepping-stone sampling analysis to infer marginal likelihoods of different multiple sequence alignment partitioning schemes and select the best one.

Four partitioning schemes of the multilocus dataset were defined a priori: by gene (byGene), splitting the substitution rates of codon positions 1+2 and 3 of each gene (byGeneCdn112) (Shapiro et al., 2006), separating the rates for each codon position (byGeneCdn123), and using the partitioning scheme suggested by PartitionFinder (Lanfear et al., 2012). We used the stepping-stone sampling method implemented MrBayes 3.2.6 (Ronquist et al., 2012) to estimate marginal likelihoods for each of the partitioning schemes by running 10 replicate Metropolis-coupled Markov chain Monte Carlo (MC^3) simulations starting from random seed trees, with 3 heated chains each, for 10^7 generations, sampling the posterior every 10^4 steps with auto-tuning of proposals enabled. The base frequencies, substitution rates and shape parameter were unlinked across partitions. The number of substitution types was set to "mixed" to allow the Markov chain to sample from the space of all possible models, assuming gamma-distributed among-site rate variation with 4 rate categories. A stepping-stone analysis (Xie et al., 2011) was performed to estimate the marginal likelihoods of each run using the ss command, with 50 steps corresponding to evenly spaced quantiles from a skewed beta distribution with alpha set to 0.4, moving from the posterior to the prior distribution, with the burninss option set to -1. The mixing, convergence and effective sample size (ESS) were evaluated for each run and parameter in tracer v1.6 http://tree.bio.ed.ac.uk/software/tracer/. Figure S7 shows parallel boxplots summarizing the results of the SS analyses based on 10 replicate runs for each partitioning scheme, as detailed in methods. The BF analysis provided overwhelming evidence in favor of the pF6p scheme, which splits codon positions across genes into 6 partitions based on their similar rates, followed by the by_gene123 scheme (ln-BF $[M_{pF6p},M_{by\_gene123}]$= 5.17975).

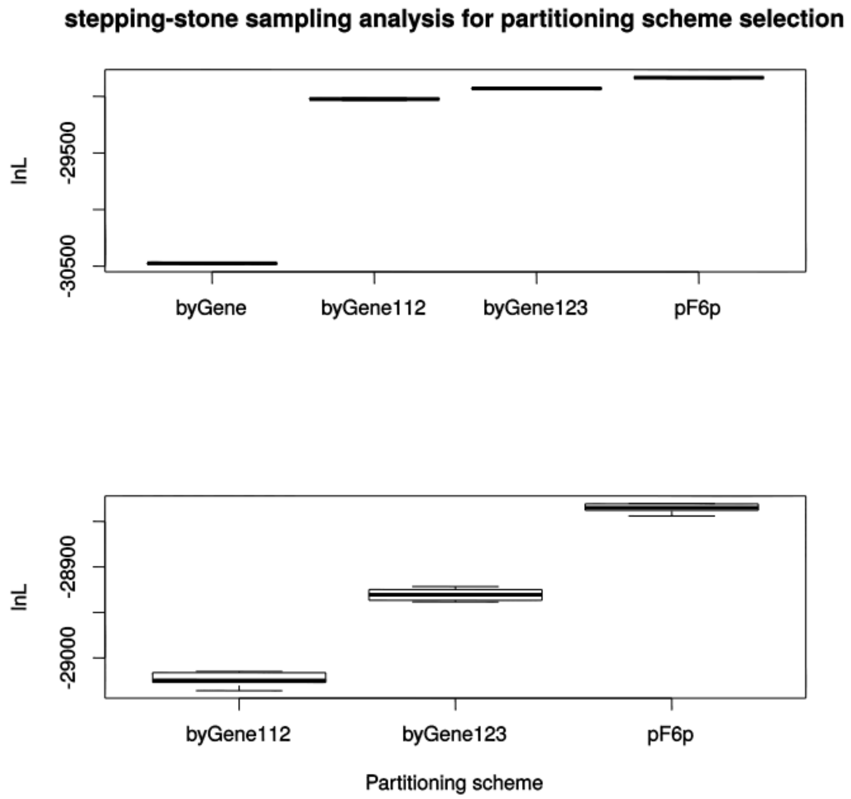

Figure S7. Parallel boxplots summarizing the results of the stepping-stone sampling analyses based on 10 replicate runs for each partitioning scheme, as detailed in methods. The graphs clearly show that the pF6p proposed by PartitionFinder (see previous section) is the best-fitting partitioning scheme of the four ones tested.

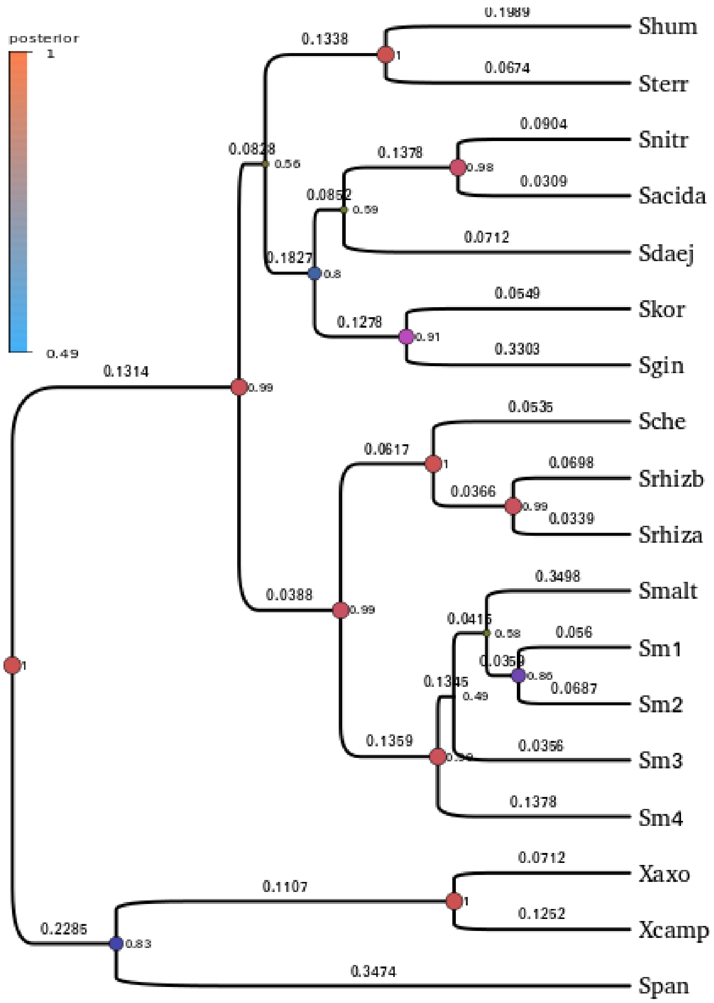

Figure S8. Consensus post-burnin (50%) Bayesian species tree from 3 replicate *BEAST runs. Most bipartitions have a significant (>0.95) posterior probability (color-coded circles at nodes), indicating that the data strongly support the topology. The exceptions are the splits subtending the Smc3 (Sm2) and *S. maltophilia* (Smalt) branches, which is most likely due to the very large population size estimated for the latter (indicated on the branches), suggesting that Smalt may contain further cryptic species. Species name abbreviations are as follows: Shum = *S. humi*; Sterr = *S. terrae*; Snitr = *S. nitrireducens*; Sacida = *S. acidaminiphila*; Sdaej = *S. daejeonesis*; Skor = *S. koreensis*; Sgin = S. *ginsengisoli*; Sche = *S. chelatiphaga*; Srhiz(ab) = *S. rhizophila*; Smalt = *S. maltophilia*; Sm1 = Smc1; Sm2 = Smc2; Sm2 = Smc3; Sm4 = Smc4; Xaxo = *Xanthomonas axonopodis*; Xcamp = *X. campestris*; Span = *S. panacihumi*.

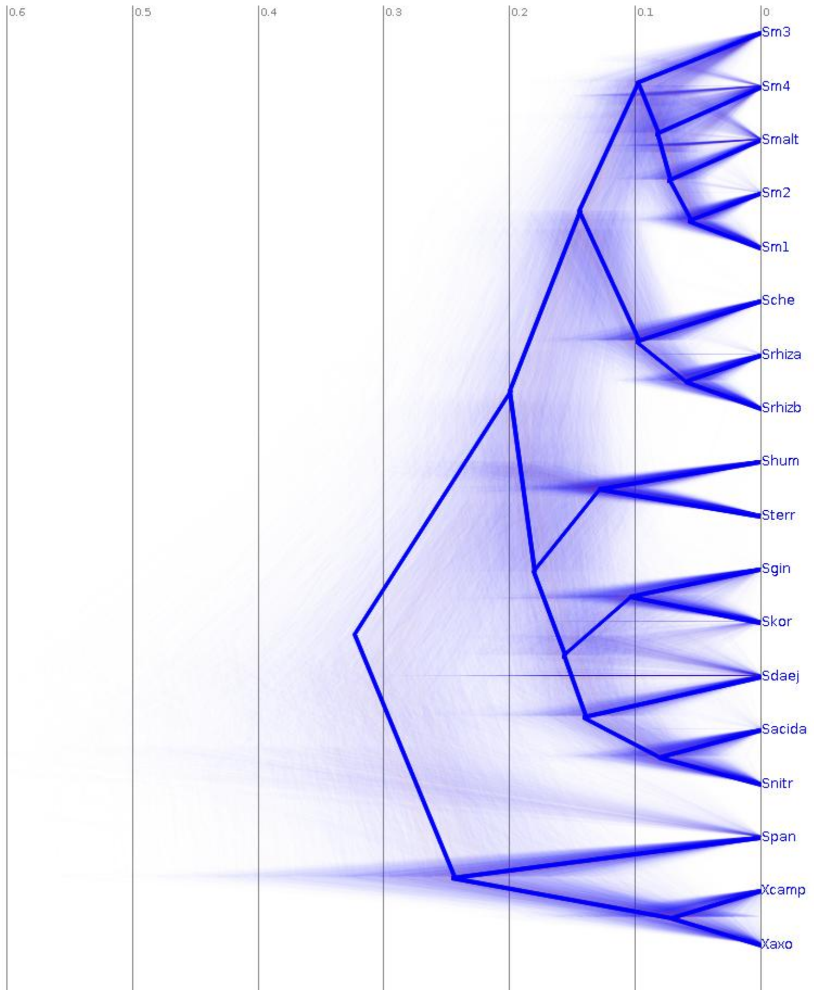

Figure S9. DensiTree (see methods) representation of the merged (3 replicate runs) post-burnin (50%) samples of *BEAST runs for the same best hypothesis shown in Fig. S8, which reveals that the source trees clearly support the separation of each species lineage, with uncertainty increasing towards the root, as expected. This tree represents the best-fitting species-delimitation hypothesis of those tested (see Figs 1A and 1B as well as Table 2 in the main text), which favors splitting the *S. maltophilia* complex into 5 species, as shown in Fig. 1B of the main text), and considering the lineages #8 and #10 as separate species (Fig. 1A).

Table S3. Summary of allele and ST assignations for Mexican isolates in the *Stenotrophomonas maltophilia* complex (Smc1=11, Smc2=15, *S. maltophilia* = 52), based on the ST profiles availabe at http://pubmlst.org/perl/bigsdb/bigsdb.pl?db=pubmlst_smaltophilia_seqdef&page=downloadProfiles&scheme_id=1, and downloaded on Nov. 18th, 2016.

**gene**	**Number of New alleles**	**New allele numbers**
atpD	24	87 to 110
gapA	27	95 to 121
guaA	38	124 to 161
mutM	43	81to 123
nuoD	29	89 to 117
ppsA	42	107 to148
recA	29	87 to115

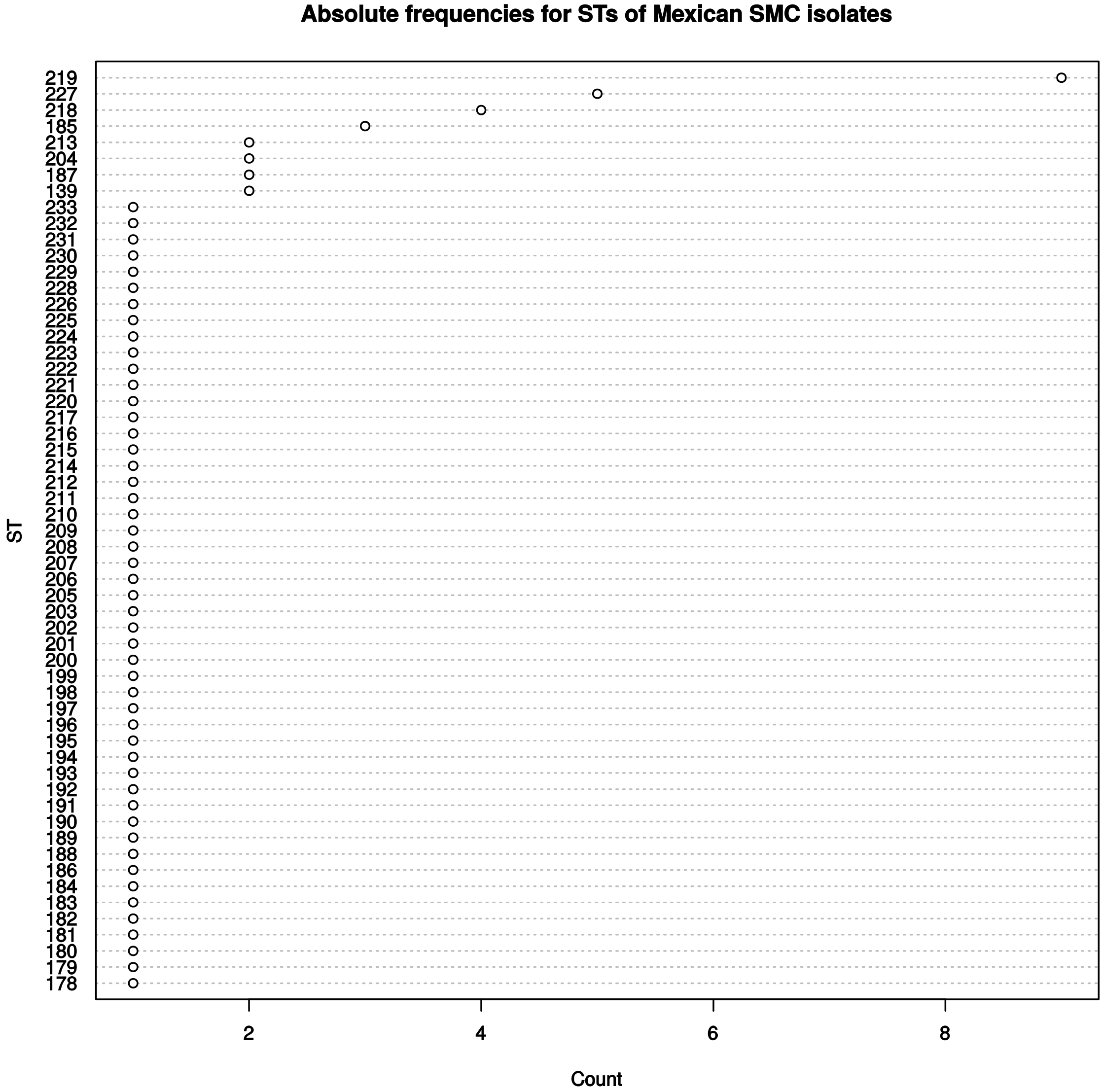

Figure S10. Cleveland dot-chart showing the absolute frequencies of the novel sequence types (STs) detected in the collection of Mexican *S. maltophilia* complex isolates. These rang from ST178 to ST233, as listed in Table S4.

Table S4. Allele and ST assignations for each of the 77 Mexican isolate from the Smc, based on the http://pubmlst.org/smaltophilia/ MLST database, which was last queried on Nov. 18th, 2016, corresponding to the database update from 2016-10-27, when it contained 663 sequences, 177 STs and 266 isolates.

strain	*atpD*	*gapA*	*guaA*	*mutM*	*nuoD*	*ppsA*	*recA*	ST
EST14A_MKIMI4_8	21	95	124	81	89	107	87	178
ESTM1D_MKCIP4_1	87	96	125	82	90	108	88	179
SAU14A_NACAZ8_4	87	97	126	83	90	109	60	180
SAU14A_NAIMI4_3	87	97	126	84	90	110	89	181
SAU14A_NAIMI4_5	88	98	127	85	91	111	90	182
SAU14Dr_NAIMI4_8	89	99	128	86	92	112	91	183
ZAC14A_NAIMI4_1	90	100	129	87	93	113	92	184
ZAC14D1_MKIMI4_2	91	95	130	88	94	114	93	185
ZAC14D1_NAIMI4_1	91	95	130	88	94	114	93	185
ZAC14D1_NAIMI4_12	91	95	130	89	94	114	93	186
ZAC14D1_NAIMI4_6	91	95	130	88	94	114	93	185
ESTM1A_MKIMI1_10	92	101	131	90	95	115	94	187
ESTM1A_MKIMI4_12	92	101	131	90	96	115	94	188
ESTM1A_MKIMI4_2	92	101	131	90	95	115	94	187
ESTM1A_MKIMI4_5a	92	101	132	90	95	115	94	189
SAU14A_NAIMI4_4	93	102	133	91	97	116	95	190
SAU14A_NAIMI4_8	93	102	133	92	97	116	95	191
SAU14Dr_NAIMI4_5	94	103	134	93	98	117	96	192
SAU14Dr_NA_5	94	103	134	93	99	117	96	193
YAU14A_MKIMI4_1	93	102	135	94	97	118	95	194
YAU14D1_LEIMI4_1	95	104	136	95	100	119	97	195
ZAC14A_NAIMI4_3	94	105	137	96	101	120	98	196
ZAC14D1_NAIMI4_9	96	104	138	97	100	119	97	197
ZAC14D2_LEIMI4_13	93	106	139	91	97	121	95	198
ZAC14D2_NAIMI4_6	97	103	140	98	102	122	99	199
ZAC14D2_NAIMI4_7	98	103	141	99	103	123	100	200
EST14D1_LEIMI4_12	74	79	142	100	87	124	101	201
EST14D1_MKIM4_1	99	107	143	101	104	125	102	202
EST14D2_LEIMI4_14	81	108	144	102	105	126	103	203
EST14D2_MKIMI4_1	74	109	145	103	87	127	101	204
EST14D2_MKIMI4_2	74	109	145	103	87	127	101	204
ESTM1A_MKCAZ16_6	100	8	146	104	4	15	104	205
ESTM1A_MKCAZ1_5	101	110	147	105	18	128	105	206
ESTM1A_MKCTX4_10a	6	111	148	18	106	129	20	207
ESTM1A_MKCTX4_10b	6	111	148	18	106	130	106	208
ESTM1A_RACAZ16_16	102	112	149	106	107	131	107	209

ESTM1D_MKCAZ16_6	3	4	110	46	6	38	58	139
ESTM1D_MKCTX4_11	102	112	149	106	107	132	107	210
SAU14A_NAIMI4_1	66	68	128	107	108	133	60	211
SAU14Da_NACAZ8_10	103	113	150	108	109	134	108	212
SAU14Da_NAIMI4_8	104	114	151	109	110	135	109	213
SAU14Dr_NACAZ8_5	43	13	52	27	111	136	17	214
SAU14Dr_NAIMI4_11	104	114	151	109	110	135	109	213
TEXM2A_MKIMI4_10	105	115	152	110	4	137	110	215
TEXM2A_MKIMI4_3	105	115	152	111	4	138	110	216
TEXM3A_MKIMI4_1	106	116	153	112	112	139	111	217
TEXM3A_NACIP1_4	102	112	154	106	107	140	107	218
TEXM3D_MKCAZ8_3	102	112	154	106	107	141	107	219
TEXM3D_MKCAZ8_7	102	112	154	106	107	140	107	218
TEXM3D_MKIMI4_2	102	112	154	113	107	141	107	220
TEXM3D_MKIMI4_3	102	112	154	114	107	141	107	221
TEXM3D_MKIMI4_4	102	112	154	106	107	141	107	219
TEXM3D_MKIMI4_5	102	112	154	106	107	141	107	219
TEXM3D_MKIMI4_6	102	112	154	106	107	141	107	219
TEXM3D_MKIMI4_7	102	112	154	106	113	141	107	222
TEXM3D_MKIMI4_8	102	112	154	106	107	141	107	219
TEXM3D_NACAZ8_1	102	112	154	106	107	141	107	219
TEXM3D_NACAZ8_2	102	112	154	115	107	141	107	223
TEXM3D_NACIP1_1	102	112	154	106	107	141	107	219
TEXM3D_NACIP1_2	102	112	154	116	107	141	107	224
TEXM3D_NACIP1_3	102	112	154	106	107	140	107	218
TEXM3D_NACIP1_4	102	112	154	106	107	140	107	218
TEXM3D_NACIP1_6	102	112	154	106	107	141	107	219
TEXM3D_NA_2	102	112	154	106	107	141	107	219
YAU14A_NACAZ8_1	106	117	155	117	114	142	112	225
YAU14A_NACAZ8_6	106	117	155	118	114	142	112	226
YAU14D1_LEIMI4_7	3	4	110	46	6	38	58	139
ZAC14D1_MKIMI4_10	81	118	156	119	72	143	113	227
ZAC14D1_MKIMI4_6	81	118	156	119	72	143	113	227
ZAC14D1_MKIMI4_8	81	118	157	119	72	143	79	228
ZAC14D1_NAIMI4_10	81	118	156	119	72	143	113	227
ZAC14D1_NAIMI4_5	81	118	156	119	72	143	113	227
ZAC14D1_NA_5	81	118	156	119	72	143	113	227
ZAC14D2_MKIMI4_1	107	119	158	120	115	144	114	229
ZAC14D2_MKIMI4_2	108	90	159	121	6	145	80	230
ZAC14D2_MKIMI4_9	109	120	158	122	116	146	115	231
ZAC14D2_NAIMI4_1	110	8	160	33	4	147	110	232
ZAC14D2_NAIMI4_3	13	121	161	123	117	148	22	233

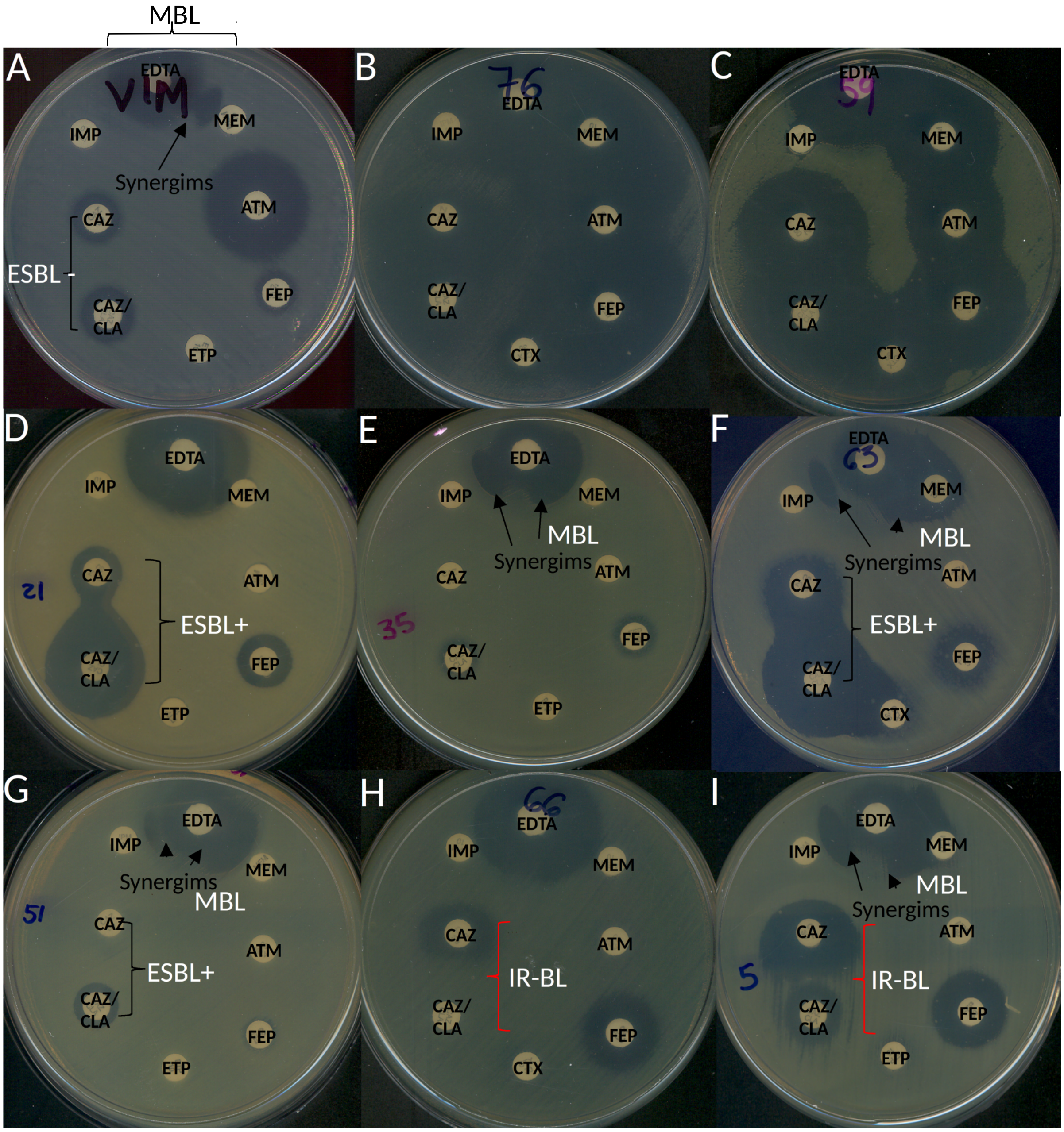

Fig. S11. Diversity of β-lactamase expression phenotypes detected among the Mexican environmental *Stenotrophomonas* isolates (B-I) using the double disk synergy (DDS) assay and the CLSI breakpoints listed in Table S2. **A**) classical MBL (VIM-2) producing phenotype expressed by a clinical *Pseudomonas aeruginosa* strain used as a control, and displaying a clear synergism between the carbapenem antibiotic meropenem (MER) and the EDTA disc. In this control strain we can also see the lack of Aztreonam (ATM) hidrolysis, as MBLs can't hydrolyze it. The lack of synergism between CAZ and CAZ / CLA reveals that this particular *P. aeruginosa* strain does not produce extended-spectrum β-lactamases (ESBL negative). **B**) A highly sensitive *S. terrae* strain EST14D2_NAIMI4_5 is shown, which is fully sensitive to all antibiotics tested (coding = 0). **C**) The picture depicts the highly sensitive Sm2 strain SAU14Dr_NAIMI4_5, sensitive to all β-lactams (no ESBL or MBL production), except to the carbapenem imipenem. **D**) The Smc2 strain ESTM1D_MKCIP4_1 displays a clear synergism between CAZ and CAZ / CLA, revealing the production of an ESBL (coding = E). **E**) Depicts the synergism

between EDTA and both carbapenems tested (IMP, MEM), indicating the production of an MBL by the S. maltophilia strain TEXM3D_MKIMI_4 (coding = M). **F,G**) Display the diversity of CAZ and CAZ / CLA synergisms observed in the ESBL-producing *S. maltophilia* strains SAU14Dr_NAIMI4_11 and AU14A_NAIMI4_1, respectively, both of which coexpress an MBL (coding = E_M. **H**) Depicts the phenotype expressed by the *S. maltophilia* strain SAU14Da_NACAZ8_10, which suggests the presence of a potentially novel clavulanate-inducible (CI) [inhibidor-resistant (IR) class A (Bush et al. 1991) or class C (AmpC)] cephalosporinase (Lister et al. 1999) (coding = CIC). **I**) The *S. maltophilia* strain ESTM1A_MXCTX4_10a also expresses a CIC, in combination with an MBL (coding = CI_M). It is interesting to note that several MBL-producing strains show sensitivity against cefepime (**F**,**I**). The phenotypes described above, were coded as indicated in parenthesis for the multiple correspondence analysis shown in the main text and in Fig. S12.

Notes on the interpretation of the DDS test.

With the arrangement of the discs shown in Fig. S11 diverse types of β-lactamases can be evidenced. The expression of an Ambler class B metallo-β-lactamase (MBL) is revealed by the synergism between EDTA with any of the carbapenems imipenem (IMP) and / or meropenem (MEM). The synergism between ceftazidime and clavulanic acid (CAZ/CLA) reveals the presence of extended-spectrum-β-lactamase (ESBL). If the zone of inhibition of Ceftazidime with clavulanate (CAZ/CLA) minus the zone of inhibition of ceftazidime (CAZ) is $\geq$ 5mm, the strain is cosidered to be ESBL positive. MBL-producing strains typically don't hydrolise aztreonam. Resistance to cefepime (FEP) suggests the presence of AmpC or ESBL.

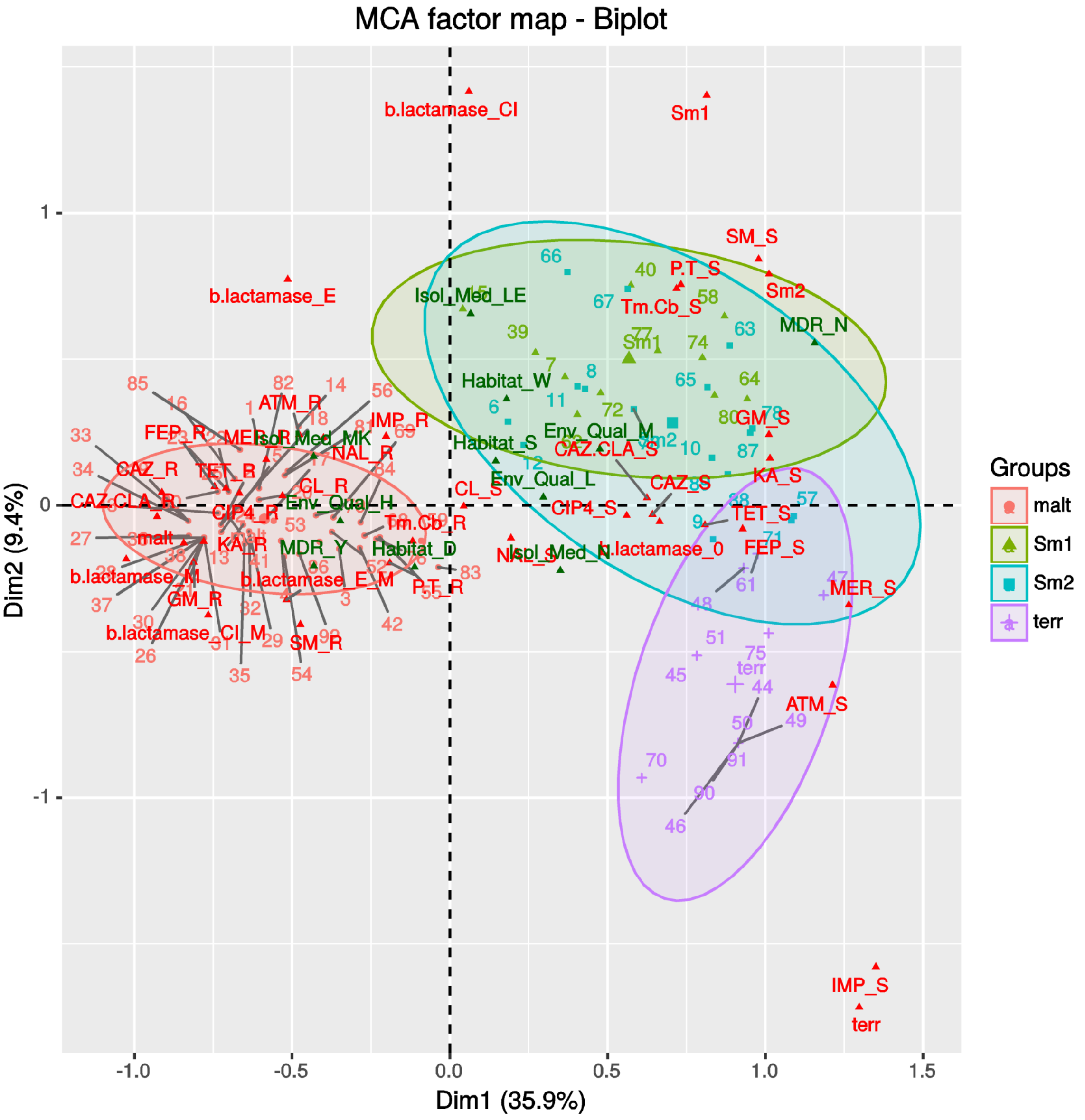

Fig. S12. Individuals-Factor biplot map resulting from the multiple correspondence analysis (MCA) of 17 active variables (red-coloured), four supplementary categorical variables (habitat, isolation medium, environmental quality and MDR status). The ellipses enclose individuals assigned to the four most abundant species (> 10 isolates) recovered from Mexican rivers, at the 95% confidence interval. Species name abbreviations are as follows: malt = *S. maltophilia*; Sm1 = Smc1; Sm2 = Smc2; terr = *S. terrae*.

A **B**

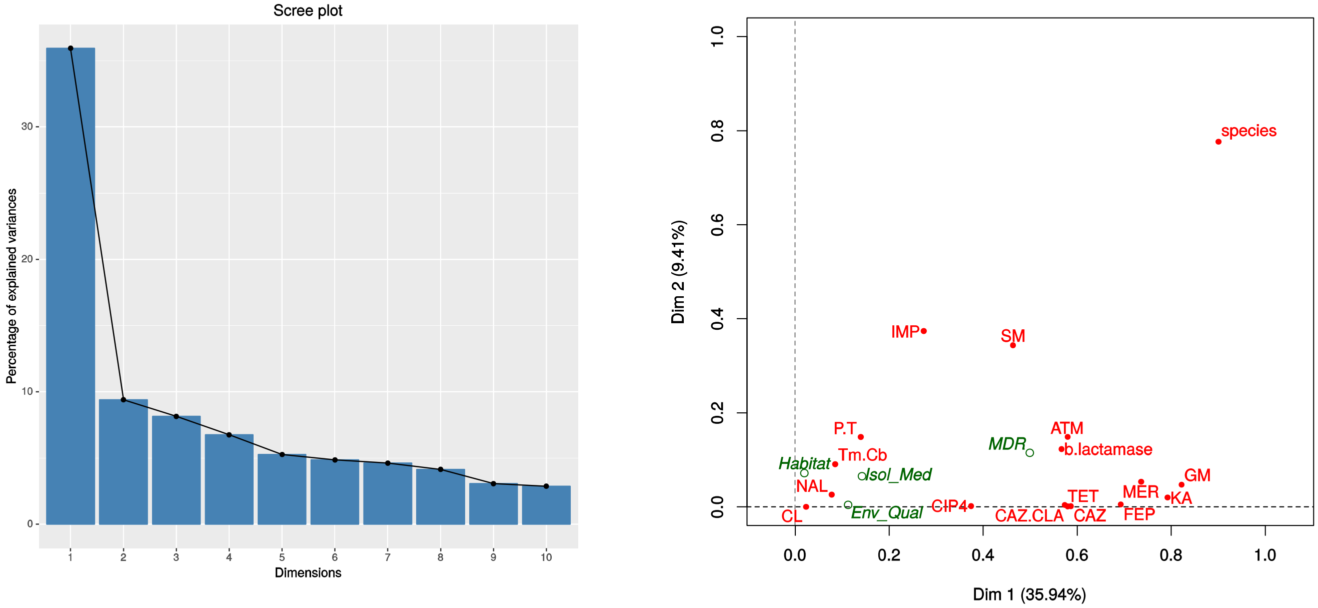

Fig. S13. Screeplot (A) for the first 10 dimensions of the MCA presented in Fig. S12 and the variable plot (B) showing their correlations with the first and second dimensions.

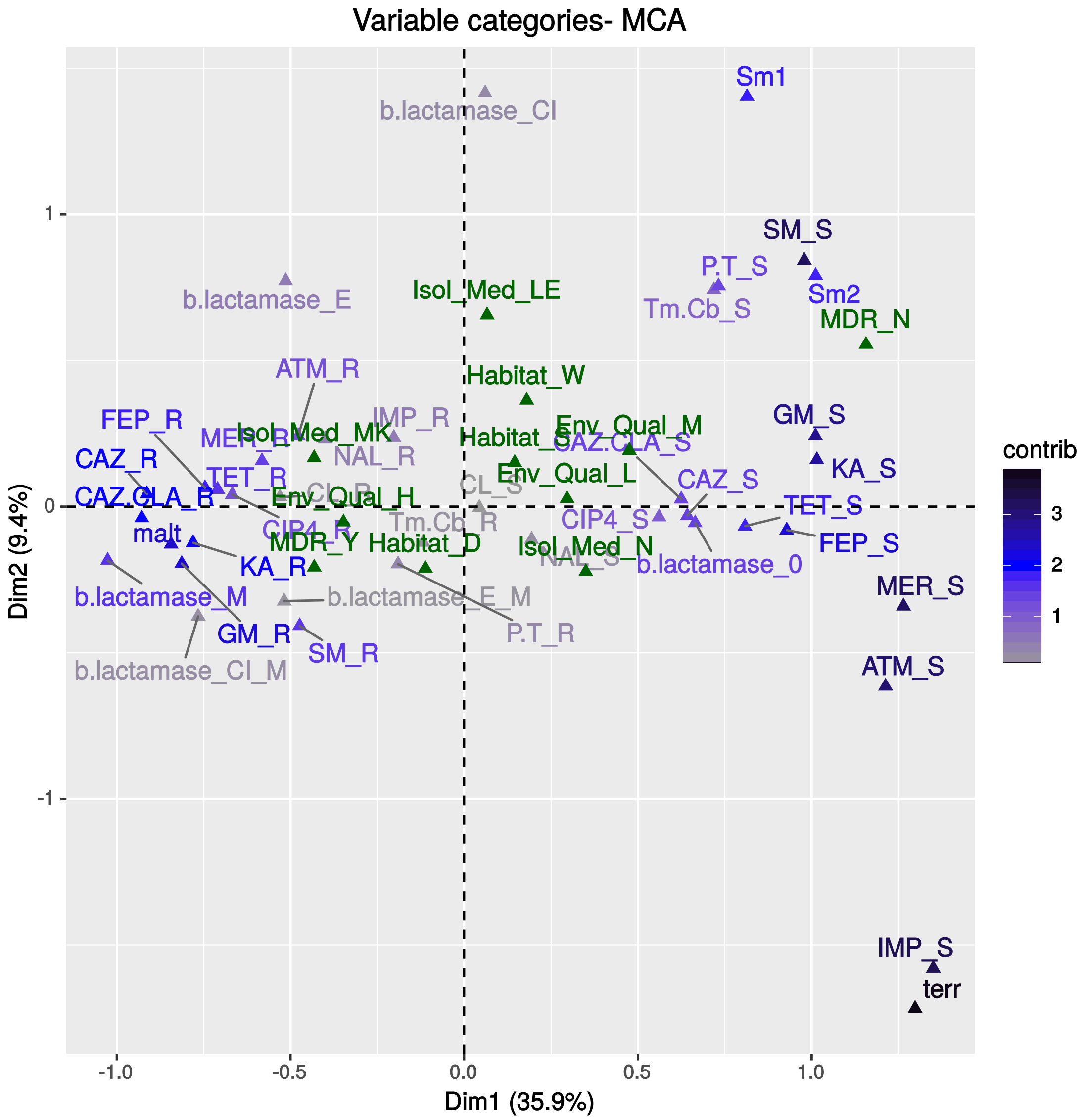

Figure S14. The Variable-categories plot, showing their contributions coded as indicated on the color scale.

Table S5. D'Agostino's test of skewness for the species-specific NumR and NumFam data.

data	skew	z	*p*-value
dfr.smalt$NumR	-0.57897,	-1.80020,	0.07184
dfr.smalt$NumFam	-0.88607,	-2.60780,	0.009112**
dfr.sm1$NumR	0.43513,	0.79391,	0.4272
dfr.sm1$NumFam	0.38420,	0.70233,	0.4825
dfr.sm2$NumR	-0.071881,	-0.152440,	0.8788
dfr.sm2$NumFam	0.57799,	1.19320,	0.2328
dfr.terr$NumR	-0.54765,	-1.04280,	0.2971
dfr.terr$NumFam	-0.74793,	-1.40340,	0.1605

Table S6. Shapiro test of normality for the total and species-specific NumR and NumFam data.

data	W	*p*-value
dfr.total.clean$NumR	0.89383	0.000001557***
dfr.total.clean$NumFam	0.86688	0.0000001215***
dfr.smalt$NumR	0.93796	0.009261**
dfr.smalt$NumFam	0.70152	0.00000000555***
dfr.smc1$NumR	0.8155	0.01505*
dfr.smc1$NumFam	0.87859	0.09985
dfr.smc2$NumR	0.94551	0.3895
dfr.smc2$NumFam	0.8386	0.007222**
dfr.terr$NumR	0.90913	0.1784
dfr.terr$NumFam	0.75584	0.002134**

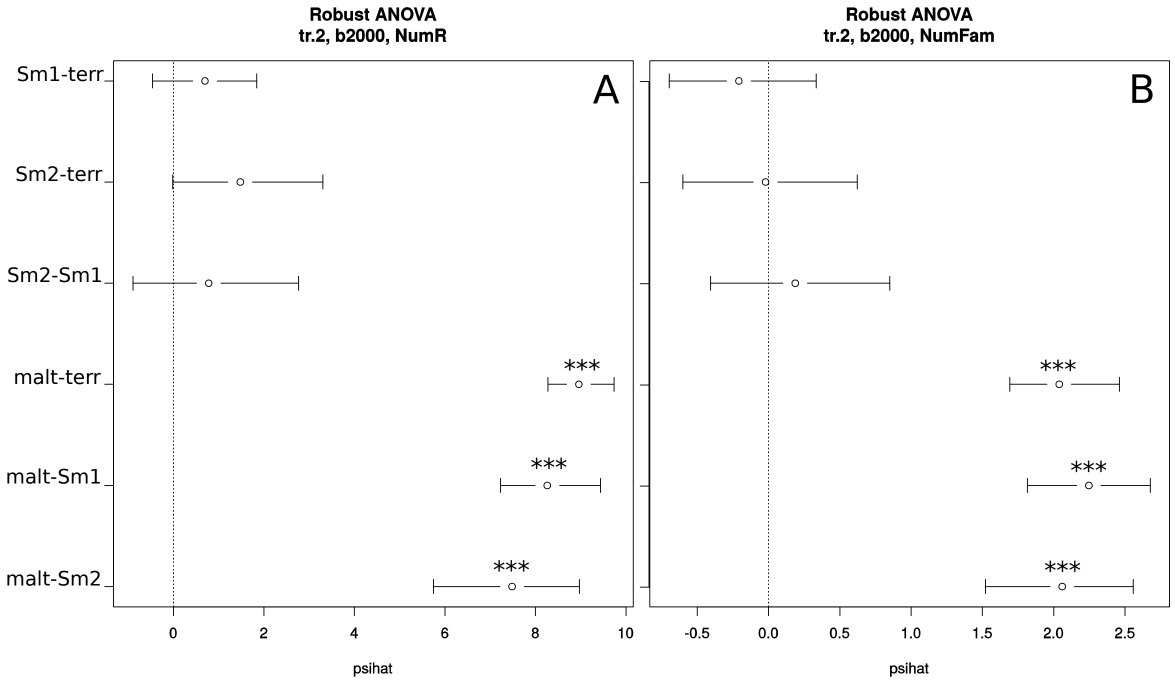

Fig. S15. Graphical display of Wilcox's *post-hoc* test for the bootstrap-t (*nboot* = 2000) version of the heteroscedastic one-way ANOVA for trimmed means (*tr* = .2) for NumR and NumFam variables as a function of species. Both the confidence intervals and the *p*-values are corrected for the number of tests. The significant contrasts are those for which the $CI_{95\%}$ does not overlap 0. Only the contrasts involving comparisons with *S. maltophilia* are highly significant ($p < 0.000001$), both for NumR (**A**) and NumFam (**B**), indicating that the former displays a significantly higher number of resistances towards a higher number of different antibiotic families than the other three species. No significant differences in resistance are found between the latter three species (See tables S7 and S8 below).

The following tables summarize the results of Wilcox's robust 1-way ANOVA with trimmed means (*tr* = .2) and bootstrap-estimated (*nboot* = 2000) sampling distribution for the trimmed means of the number of antibiotic resistances (NumR) and number of resistance families (NumFam).

Table S7. Wilcox's *post-hoc* analysis for the analysis of NumR by t1waybt (*tr* =.2; boot=2000). Test statistic: 149.9521, *p*-value: 0. Variance explained 0.652. Effect size 0.808

**comparisons**	**psihat**	**ci.lower**	**ci.upper**	***p*-value**
**malt-Sm2**	7.48214	5.76339	9.00000	0.0000
**malt-Sm1**	8.26136	7.17330	9.39773	0.0000
**malt-terr**	8.95833	8.27431	9.72222	0.0000
**Sm2-Sm1**	0.77922	-1.01299	2.72727	0.5210
**Sm2-terr**	1.47619	-0.07937	3.30159	0.1150
**Sm1-terr**	0.69697	-0.43434	1.88889	0.3055

Table S8. Wilcox's *post-hoc* analysis for the analysis of NumFam by t1waybt (*tr* =.2; boot=2000) for NumFam. Test statistic: 51.1979; *p*-value: 0. Variance explained 0.723. Effect size 0.85

**comparisons**	**psihat**	**ci.lower**	**ci.upper**	***p*-value**
**malt-Sm2**	2.37054	1.70536	2.89286	0.0000
**malt-Sm1**	2.65625	1.96307	3.10795	0.0000
**malt-terr**	2.10069	1.67708	2.63889	0.0000
**Sm2-Sm1**	0.28571	-0.49351	1.02597	0.6460
**Sm2-terr**	-0.26984	-0.87302	0.55556	0.6165
**Sm1-terr**	-0.55556	-1.07071	0.24242	0.2625

Supplementary References

Bush K., Flamm R.K., Ohringer S., Singer S.B., Summerill R., Bonner D.P. (1991). Effect of clavulanic acid on activity of beta-lactam antibiotics in *Serratia marcescens* isolates producing both a TEM beta-lactamase and a chromosomal cephalosporinase. *Antimicrob. Agents Chemother*. **35**:2203-8.

Camacho, C., Coulouris, G., Avagyan, V., Ma, N., Papadopoulos, J., Bealer, K., and Madden, T.L. (2009) BLAST+: architecture and applications. *BMC Bioinformatics* **10**: 421.

CLSI (2016) *Clinical and Laboratory Standards Institute (CLSI) Performance Standards for Antimicrobial susceptibility testing.* Wayne, Pennsylvania Clinical and Laboratory Standards Institute.

Cole, J.R., Chai, B., Farris, R.J., Wang, Q., Kulam-Syed-Mohideen, A.S., McGarrell, D.M. et al. (2007) The ribosomal database project (RDP-II): introducing myRDP space and quality controlled public data. *Nucleic Acids Res.* **35**: 169-172.

Darriba, D., Taboada, G.L., Doallo, R., and Posada, D. (2012) jModelTest 2: more models, new heuristics and parallel computing. *Nat. Methods* **9**: 772.

Ewing, B., and Green, P. (1998) Base-calling of automated sequencer traces using phred. II. Error probabilities. *Genome Res.* **8**: 186-194.

Guindon, S., Dufayard, J.F., Lefort, V., Anisimova, M., Hordijk, W., and Gascuel, O. (2010) New algorithms and methods to estimate maximum-likelihood phylogenies: assessing the performance of PhyML 3.0. *Syst. Biol.* **59**: 307-321.

Lanfear, R., Calcott, B., Ho, S.Y., and Guindon, S. (2012) Partitionfinder: combined selection of partitioning schemes and substitution models for phylogenetic analyses. *Mol. Biol. Evol.* **29**: 1695-1701.

Larkin, M.A., Blackshields, G., Brown, N.P., Chenna, R., McGettigan, P.A., McWilliam, H. et al. (2007) Clustal W and Clustal X version 2.0. *Bioinformatics* **23**: 2947-2948.

Lister PD, Gardner VM, Sanders CC (1999). Clavulanate induces expression of the *Pseudomonas aeruginosa* AmpC cephalosporinase at physiologically relevant concentrations and antagonizes the antibacterial activity of ticarcillin. A*ntimicrob Agents Chemother.* 1999 Apr;**43**(4):882-889.

Ronquist, F., Teslenko, M., van der Mark, P., Ayres, D.L., Darling, A., Hohna, S. et al. (2012) MrBayes 3.2: efficient Bayesian phylogenetic inference and model choice across a large model space. *Syst. Biol.* **61**: 539-542.

Shapiro, B., Rambaut, A., and Drummond, A.J. (2006) Choosing appropriate substitution models for the phylogenetic analysis of protein-coding sequences. *Mol. Biol. Evol.* **23**: 7-9.

Xie, W., Lewis, P.O., Fan, Y., Kuo, L., and Chen, M.H. (2011) Improving marginal likelihood estimation for Bayesian phylogenetic model selection. *Syst Biol* **60**: 150-160.
